# Correlation between degree of hallux valgus and kinematics in classical ballet: A pilot study

**DOI:** 10.1371/journal.pone.0231015

**Published:** 2020-04-06

**Authors:** Haruka Seki, Akito Miura, Nahoko Sato, Jun Yuda, Toshiko Shimauchi

**Affiliations:** 1 Japan Ballet Educational Association, Tokyo, Japan; 2 Department of Movement Sciences, Japan Women’s College of Physical Education, Tokyo, Japan; 3 Faculty of Human Sciences, Waseda University, Saitama, Japan; 4 Department of Physical Therapy, Faculty of Rehabilitation Science, Nagoya Gakuin University, Aichi, Japan; The Wingate College of Physical Education and Sports Sciences at the Wingate Institute, IL, ISRAEL

## Abstract

Hallux valgus is a serious medical concern for classical ballet dancers. Although it is well-known that progression of hallux valgus is related to inappropriate movement techniques in classical ballet, the kinematic relationship between the degree of hallux valgus and ballet techniques has not been substantiated. To develop proper training methods that prevent progression of hallux valgus, this study aimed to investigate the relationship between the degree of hallux valgus and movement techniques in classical ballet. Seventeen female classical ballet dancers at the advanced college-level participated in this study. Kinematic analysis of standing and plié in the first position was conducted via video capture technique. The Pearson product-moment correlation analysis was performed to examine the degree of hallux valgus and the following three kinematic variables: (1) the extent to which turnout is forced by other joints in the lower extremity than the hip joint, (2) the direction difference between the knee and toe in the transverse plane, and (3) the pelvis obliquity angle. Among these kinematic variables, we found a significant correlation between the hallux valgus angle and the pelvis obliquity angle during plié (*P* = .045). The greater the hallux valgus angle, the greater the retroversion of the pelvis, a result which was contrary to our prediction. We present the first evidence that the degree of hallux valgus correlates with kinematics in a very basic technique of classical ballet.

## Introduction

Hallux valgus is a foot deformity characterized by malalignment of the first intermetatarsal and metatarsophalangeal joints, usually progressing irreversibly over a long period [1–4]. In conventional clinical practice, hallux valgus is diagnosed when the hallux valgus angle (i.e., the angle between the longitudinal axes of the proximal phalanx of the great toe and the first metatarsophalangeal bone) is greater than 15 degrees [4]. Etiological studies of hallux valgus have revealed that it results from complex factors, including proximal joint malalignment [5, 6], hypermobility [7–10], footwear [11], inheritance [12], and/or inappropriate movement technique [13]. Morbidity of hallux valgus is reported to be higher in dancers of various dance styles whose basic techniques are based on classical ballet [6]. It is a serious medical concern, as it affects dancers’ performance and career.

To the best of our knowledge, however, there is no study that substantiates the kinematic relationship between hallux valgus and ballet technique. If hallux valgus were related with ballet techniques [13], it can be expected that correlations would be found between the degree of hallux valgus and kinematics during the execution of ballet techniques. Furthermore, considering that hallux valgus is a deformity of foot that supports the whole body weight in contact with the ground, it is possible that hallux valgus would affect the kinematics of the entire body.

One of the inappropriate movement techniques in classical ballet that is said to exacerbate injuries, including hallux valgus, is forced turnout [6, 13–15]. Appropriate turnout should be accomplished by external rotation of hip joint [16]. When the angle of hip external rotation is insufficient, turnout is often augmented by other joints in the lower extremity and lumbar region along the kinematic chain [15, 17, 18]. In such forced turnout, there is hyperpronation of subtalar joints, called “rolling in.”[13, 15, 19] This increases force on the first metatarsophalangeal in the direction of abduction, possibly leading to progression of hallux valgus. Therefore, we expected that movement kinematics that characterize or associate with such forced turnout would be correlated with the degree of hallux valgus.

We investigated this correlation during static and dynamic basic ballet techniques (standing and plié in the first position). This is because if such correlation is found in these basics, it can be generalized to almost all ballet techniques based on these basics. Three hypotheses were examined for the correlation: first, dancers with a greater extent of forced turnout would show a greater hallux valgus angle; second, especially during plié, dancers with a greater hallux valgus would show a greater angle difference in the direction of the knee and that of the toe (i.e., knee-foot alignment in the transverse plane), and this is also thought to be associated with forced turnout [16]; and third, dancers with a greater hallux valgus angle will demonstrate more anteversion of the pelvis by considering the following two points: (1) lumbar lordosis, that is, anteversion of the pelvis, increases in order to force turnout through the kinematic chain [15] and (2) Khamis and Yizhar [20] demonstrated that hyperpronation of the subtalar joints made by an inclined slope leads to anteversion of the pelvis.

These correlational relationships, if exist, result in further better understanding of the foot-body relationship in classical ballet and contribute to developing the proper training methods preventing progression of hallux valgus. Therefore, this study aimed to investigate the relationship between the degree of hallux valgus and alignment of the lower extremity and pelvis in both static and dynamic turnout conditions.

## Materials and methods

### Participants

Among the 82 sophomore students who majored in dance at Japan Women's College of Physical Education, we recruited 19 students (19–20 years old) who were available out of the 20 students in the advanced ballet class. They specialized in classical ballet, and had had experience of classical ballet for 10 years or more at the time of this experiment. Because we missed the data of two participants due to technical problems, we excluded these participants from the subsequent analysis. The hallux valgus angle of the weight-bearing barefoot while standing in a parallel stance was measured at the medial contour of the right foot, which is reported to be in agreement with radiographic measurement (interclass correlation coefficients: 0.81) [21]. There were nine students with hallux valgus and eight without hallux valgus according to a criterion proposed by the previous study [12]. This study was in accordance with the Declaration of Helsinki and was approved by the Ethics Committee of Japan Women's College of Physical Education. Informed consent for their participation was obtained from all participants prior to the experiment.

### Experimental tasks

**Fig 1:**
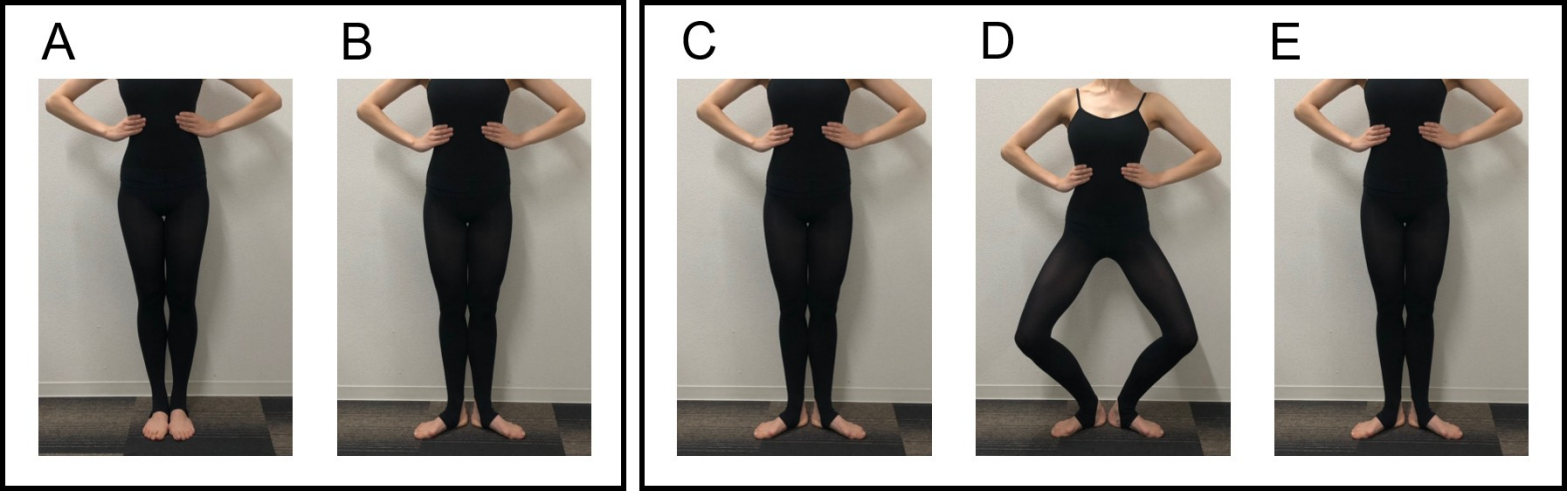
Experimental task for the (A, B) standing and (C, D, E) plié conditions. For the standing condition, participants were first instructed to stand in the sixth position (A), to change to the first position (B), and to maintain the position for a few seconds. For the plié condition, they were instructed to stand in the first position (C), to flex the knees for 1 s, to stay in the position for 1 s (D), and to extend the knees for 1 s to the first standing position (E) according to 1 Hz metronome beat. Durations for kinematic analysis were the time of (B) for standing condition and that of (D) for plié condition (please see *Data analysis* for criteria in detail).

As experimental tasks, we used standing and plié in the first position (Fig 1). This is because the correlation between the kinematics and hallux valgus can be observed not only in static posture but also during movement. In the standing condition, participants were first instructed to stand in the sixth position (Fig 1A), to change to the first position, and to maintain the position for a few seconds (Fig 1B). In the plié condition, they were instructed to perform a plié in the first position according to 1 Hz metronome beat. They were instructed to stand in the first position (Fig 1C), to flex their knees for 1 s, to stay in the position for 1 s (Fig 1D), and to extend the knees for 1 s to the first standing position (Fig 1E). In both conditions, they were instructed to put their hands on the sides of their body in order not to impede the motion of the pelvis. One trial for each condition was captured.

### Data acquisition

The 3D coordinates of participants’ bodies were measured by video analysis in the same way as previous studies [22–24]. Four digital video cameras (HC-V300M, Panasonic, Japan; HDR-HC7, SONY, Japan; DMX-HD2000, SANYO, Japan) were located around the participants. The video was recorded at 60 fields per second and at 1/1000 sec of exposure time. The four cameras were synchronized by a synchronizer with LED light (TS-166, DKH, Japan). The lights (VL-302, LPL, Japan) were attached to each camera in order to increase visibility of reflective markers on the participants. Right leg alignment was examined because both legs were assumed to be equivalent for our task, which should be confirmed in a future study. Retro-reflective markers were attached to both sides of the anterior superior iliac spine (ASIS), the right posterior superior iliac spine (PSIS) and the right leg: the medial and lateral femoral condyles, the medial and lateral malleoli, the medial side of the first metatarsophalangeal joint (MT1), and the lateral side of fifth metatarsophalangeal joint (MT5).

The 3D coordinates of the reflective markers were calculated by the DLT method [25] using movement-analysis software (Frame-Dias IV, DKH Inc., Japan). The coordinates of the hip joint center (HJC) were obtained by manual digitizing. The coordinates were filtered with a fouth-order Butterworth digital low pass filter at cut-off frequency 15 Hz, determined by residual analysis [26].

The pelvis, thigh, shin, and foot coordinate systems were defined as per a previous study [27]. The origin of pelvis coordinate systems was set at the midpoint between the ASISs, and a z-axis (Z_pelvis_) was along the line of the left ASIS to the right one (positive being right). A y-axis (Y_pelvis_) was computed using the cross-product of the Z_pelvis_ and the vector from the right PSIS to the origin (positive being superior). An x-axis (X_pelvis_) was orthogonal to the y-z-plane (positive being anterior).

The origin of thigh coordinate system was set at the mid-point between the medial and lateral femoral condyles and a y-axis (Y_thigh_) was along the line from the origin to the right HJC (positive being superior). An x-axis (X_thigh_) was computed using the cross product of the Y_thigh_ and the vector from the medial femoral condyles to the lateral one (positive being anterior). A z-axis (Z_thigh_) was orthogonal to the x-y-plane (positive being right).

The origin of the shin coordinate system was set at the mid-point between the medial and lateral malleoli and a y-axis (Y_shin_) was along the line from the origin to the mid-point between the medial and lateral femoral condyles (positive being superior). An x-axis (X_shin_) was computed using the cross product of the Y_shin_ and the vector from the medial malleolus to the lateral malleolus (positive being anterior). A z-axis (Z_shin_) was orthogonal to the x-y-plane (positive being right).

The origin of the foot coordinate system was set at the mid-point between the medial and lateral malleoli and an x-axis (X_foot_) was along the line from the origin to the mid-point between the MT1 and the MT5 (positive being anterior). A y-axis (Y_foot_) was computed using the cross product of the vector from the MT1 to the MT5 and the X_foot_ (positive being superior). A z-axis (Z_foot_) was orthogonal to the x-y-plane (positive being right).

### Data analysis

We proposed three hypotheses that the degree of hallux valgus would be correlated with (1) the extent to which the turnout is forced by inappropriate joints, (2) the knee-foot alignment in the transverse plane, and (3) the pelvis obliquity angle. The extent to which the turnout is forced was defined by the below-hip contributors to turnout, that is, the percentage of the sum of external rotation angle of knee and ankle relative to the total turnout angle. If the value is 0%, this means that turnout is accomplished only by hip external rotation. If the value is 100%, this means that turnout is forced by the knee and ankle joints without hip external rotation. The total turnout angle was calculated as the angle between X_pelvis_ and X_foot_ within the plane perpendicular to the laboratory vertical axis [27]. The hip, knee, ankle external rotation angles were defined as the angle between two consecutive anteroposterior vectors within the plane perpendicular to the longitudinal axis of the proximal segment. For example, the hip external rotation angle was defined as the angle between X_pelvis_ and X_thigh_ within the plane perpendicular to the Y_pelvis_. The pelvis obliquity angle was defined as the angle between the Y_pelvis_ and the laboratory vertical axis within the plane perpendicular to the Z_pelvis_ (the greater value means anteversion). The duration of analysis was determined as follows: for the standing condition, the phase selected for analysis was that in which the total turnout angle was in the largest 5%, where 0% represents the smallest turnout angle (i.e., the sixth position before turnout) (Fig 1A) and 100% represents the largest turnout angle (Fig 1B). For the plié condition, the phase selected for analysis was that in which the knee flexion angle was in the largest 5%, where 0% represents the smallest knee flexion angle (Fig 1C and 1E) and 100% represents the largest knee flexion angle (Fig 1D). The knee flexion angle was defined as the angle between Y_thigh_ and Y_shin_ within the plane perpendicular to the Z_thigh_. All kinematic parameters are presented as mean of the values for these analysis durations.

## Results

Hallux valgus angles and kinematic values for both conditions were summarized in Table 1.

**Table 1:** Hallux valgus angles and kinematic values for both conditions.

			Experimental task: Standing					Experimental task: Plié				
		Hallux valgus angle (deg)	External rotation angle (deg)			Extent to which the turnout is forced (%)	Pelvis obliquity angle (deg)	External rotation angle (deg)			Extent to which the turnout is forced (%)	Pelvis obliquity angle (deg)
	Dancer	Hip	Knee	Ankle	Hip	Knee	Ankle
Dancers without the hallux valgus	1	10	43.4	12.8	8.1	32.4	13.0	57.3	15.8	-2.6	18.8	12.6
	2	15	34.4	28.7	8.4	51.8	15.2	39.1	0.3	24.7	39.0	15.3
	3	15	43.2	32.8	8.4	48.8	11.2	47.6	17.2	10.6	36.8	20.0
	4	11	36.8	40.1	2.6	53.7	17.1	47.3	29.1	4.4	41.5	17.5
	5	10	42.9	13.8	7.2	32.8	15.7	58.3	9.1	7.1	21.7	16.3
	6	13	26.2	34.4	-2.9	54.6	15.7	37.5	22.9	-6.6	30.4	17.9
	7	15	30.2	7.0	22.8	49.7	24.1	43.7	-6.5	23.5	28.0	23.8
	8	15	31.9	21.3	6.1	46.2	25.5	35.3	8.5	14.3	39.2	23.0
Dancers with the hallux valgus	9	29	15.1	28.1	10.8	72.0	18.0	19.9	19.2	3.3	53.1	15.6
	10	35	21.1	31.9	5.3	63.8	14.7	41.4	16.4	0.3	28.8	12.8
	11	30	30.5	33.8	0.0	52.6	10.0	42.3	21.2	1.0	34.4	12.9
	12	25	50.6	26.6	5.4	38.8	17.4	45.4	12.2	7.6	30.4	17.5
	13	20	31.0	29.6	-0.2	48.7	17.1	46.6	17.2	3.5	30.8	16.1
	14	25	33.9	30.9	-3.5	44.7	13.9	40.5	20.0	5.7	38.9	11.1
	15	30	42.2	23.5	5.7	40.9	16.2	46.0	19.0	7.7	36.8	16.0
	16	35	40.2	21.1	-2.3	31.8	14.4	44.3	10.8	-1.0	18.0	14.4
	17	29	31.2	24.6	10.6	53.0	15.4	42.4	17.8	3.7	33.6	11.0

### Correlation between hallux valgus angle and the extent to which turnout is forced

**Fig 2:**
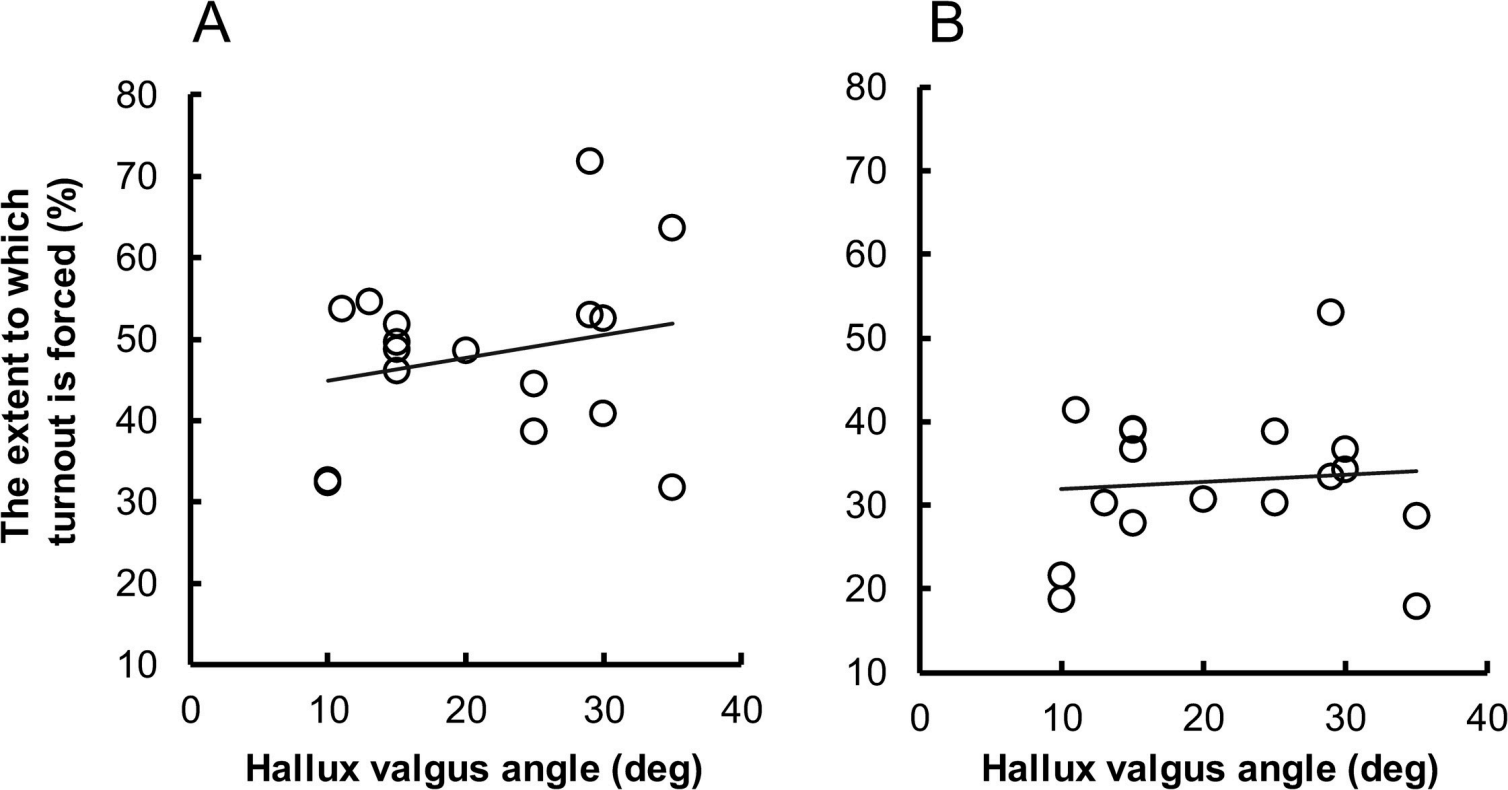
The scatter plots of the hallux valgus angle versus the extent to which the turnout is forced. During (A) standing and (B) plié in the first position. The extent to which the turnout is forced is measured by the below-hip contributors (i.e., the percentage of sum of external rotation angle of knee and ankle relative to the total turnout angle).

Fig 2 shows a scatter plot of the hallux valgus angle versus the extent to which the turnout is forced (i.e., the percentage of sum of external rotation angle of knee and ankle relative to the total turnout angle). The Pearson product-moment correlation analysis revealed no significant correlation between these variables for both the first position standing (*r* = 0.231, *P* = .372) and pile (*r* = 0.088, *P* = .737).

### Correlation between hallux valgus angle and knee-foot alignment in the transverse plane

**Fig 3:**
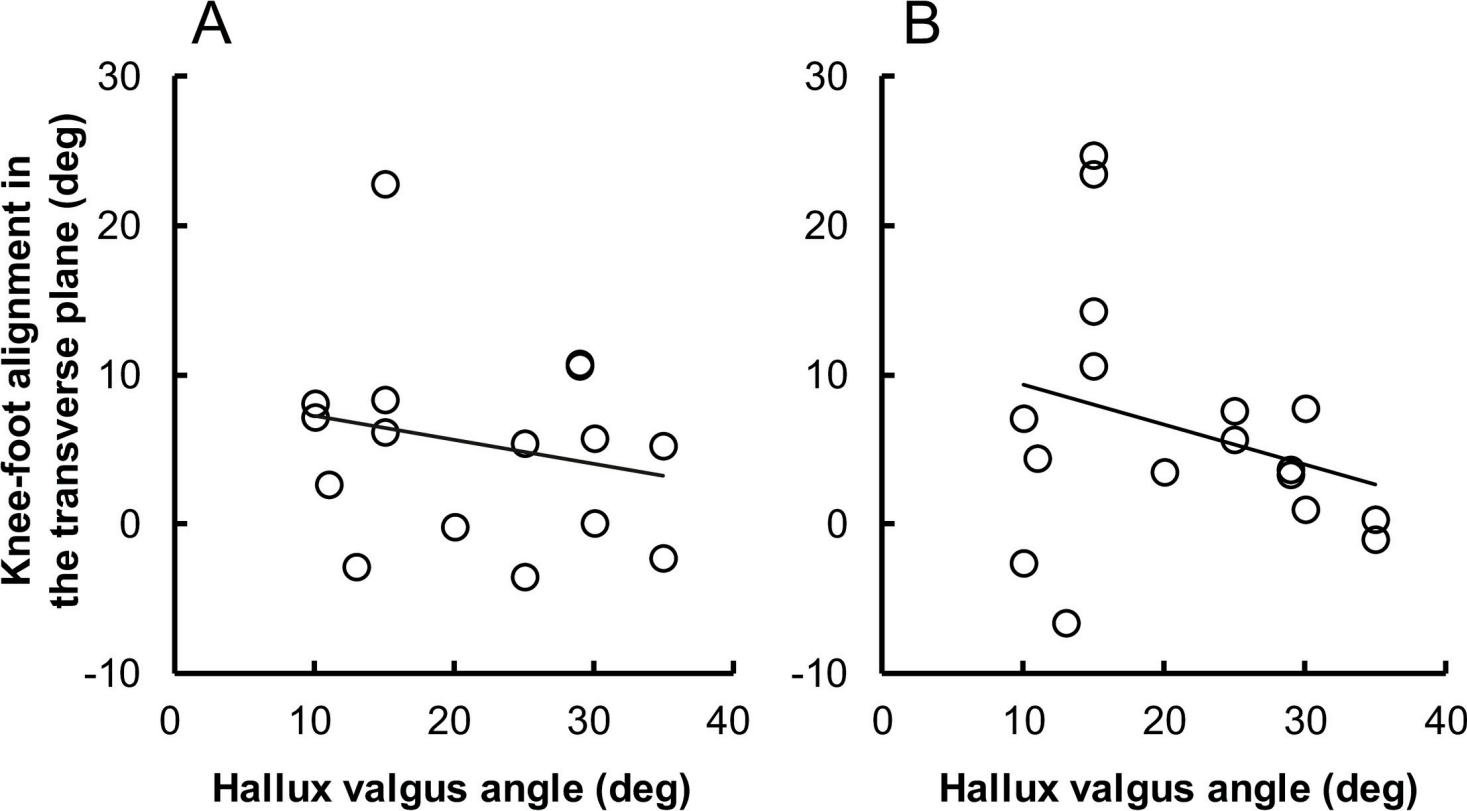
The scatter plot of the hallux valgus angle versus the knee-foot alignment in the transverse plane. During (A) standing and (B) plié in the first position.

Fig 3 shows a scatter plot of the hallux valgus angle versus the knee-foot alignment in the transverse plane. The Pearson product-moment correlation analysis revealed no significant correlation between these variables for both the first position standing (*r* = -0.218, *P* = .400) and plié (*r* = -0.284, *P* = .269).

### Correlation between hallux valgus angle and pelvis obliquity angle

**Fig 4:**
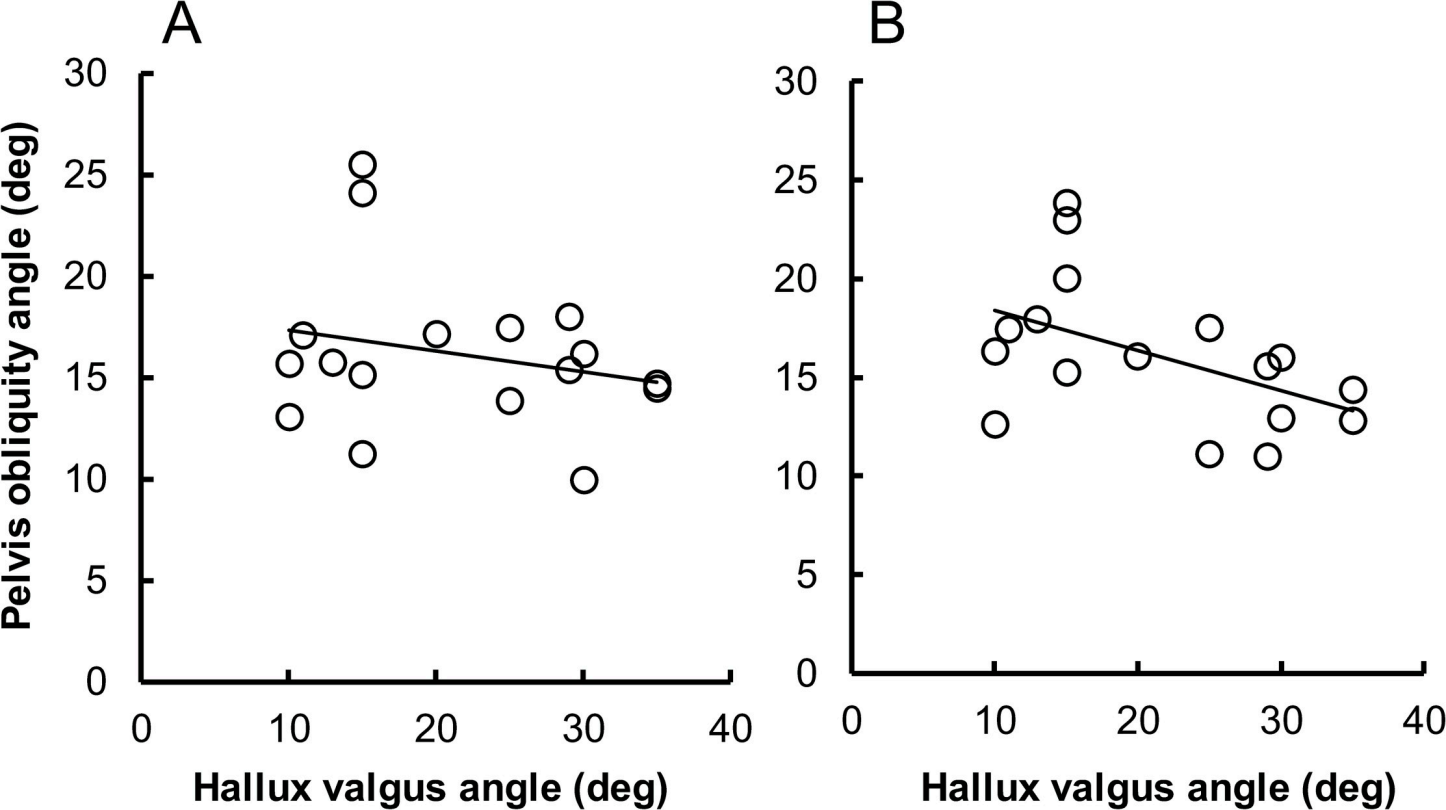
The scatter plot of the hallux valgus angle versus the pelvis obliquity angle. During (A) standing and (B) plié in the first position. The greater pelvis obliquity angle means anteversion. The Pearson product-moment correlation analysis revealed significant correlation between these variables only for plié (*r* = -0.491, *P* = .045).

Fig 4 shows a scatter plot of the hallux valgus angle versus the pelvis obliquity angle (the greater value means anteversion). The Pearson product-moment correlation analysis revealed a significant correlation between these variables for plié (*r* = -0.491, *P* = .045), but not for the first position (*r* = -0.236, *P* = .361). These results indicate that the greater the hallux valgus angle, the greater the retroversion of the pelvis during plié.

## Discussion

The purpose of this study was to investigate the relationship between the degree of hallux valgus and kinematics in ballet techniques. We hypothesized that the degree of hallux valgus would correlate with kinematic variables during execution of ballet techniques. We found significant correlations between the degree of hallux valgus and the pelvis obliquity angle during plié. The other kinematic variables did not correlate with hallux valgus angle.

Contrary to our expectation, we did not find significant correlation between the hallux valgus angle and the extent to which turnout is forced (i.e., the percentage of sum of external rotation angle of knee and ankle relative to the total turnout angle) (Fig 2). Similarly, we did not find a significant correlation between the hallux valgus angle and the knee-foot alignment in the transverse plane (Fig 3). These kinematic characteristics are thought to be inappropriate ballet techniques that can exacerbate injuries, including hallux valgus [6, 13, 14, 16] that is now common knowledge among ballet dancers and educators. Therefore, there is a possibility that the participants with hallux valgus might have performed such inappropriate ballet techniques at earlier stages of their careers, but had modified these inappropriate ballet techniques at the time of the experiment, considering their relatively advanced level of ballet (i.e., advanced college-level).

We found a significant correlation between the hallux valgus angle and the pelvis obliquity angle during plié (Fig 4B). The greater the hallux valgus angle, the greater the retroversion of the pelvis. This result was also contrary to our hypotheses. Although it is well known that the anteversion of the pelvis increases during forced turnout [15], our findings suggest that the participants had modified the forced turnout (Figs 2 and 3). Therefore, we believe this well-known kinematic chain was not observed in our participants. We made the hypothesis based also on a previous study where induced hyperpronation of the subtalar joints by an inclined slope led to the anteversion of the pelvis in a kinematic chain reaction [20]. In that study, the participants stood on the inclined slope in a relaxed and natural position. In this stance, the induced hyperpronation leads to internal rotation of the tibia and thigh, and this leads to the anteversion of the pelvis. On the other hand, our findings suggest that the hyperpronation of subtalar joints, thought to be correlated with the degree of hallux valgus, with the lower extremities externally rotated and with the knees flexed (i.e., plié with turnout), would lead to retroversion of the pelvis through the kinematic chain.

One limitation of our study is that we could not substantiate the expected correlation between hyperpronation of the subtalar joints and the degree of hallux valgus. This is because we could not quantify foot pronation, which requires higher resolution of the video analysis than was available to us. A previous study demonstrated using 3D multi-segment foot model that female college dance students pronate their feet via hindfoot eversion and midfoot abduction during turnout compared with a natural stance [19]. Although they did not report the relationship between the extent of pronation and degree of hallux valgus, their proposed model that can measure foot motion on a segment basis is beneficial to investigate this relationship in a future study.

Despite this limitation, we confirmed that the degree of hallux valgus correlated with the pelvis obliquity angle during basic ballet technique. Because plié in the first position is the very basic for other ballet techniques, this correlation can be observed in the other ballet techniques based on this basic technique. Especially for more dynamic techniques, this correlation should be more prominent. We believe that accumulation of knowledge regarding the kinematic relationship between the hallux valgus and ballet technique contributes to develop proper training methods that prevents a progression of hallux valgus.

## Conclusions

This study reported the first evidence that the degree of hallux valgus relates kinematics in basic ballet techniques. We would like to emphasize that because we found this correlation in the very basic techniques in classical ballet, hallux valgus would affect almost all ballet techniques that are based on this basic technique. Further research is needed to clarify the effect of hallux valgus on more complex ballet techniques. This would contribute to the development of treatment and prevention of hallux valgus in ballet dancers.
